# Three new entomopathogenic fungal species isolated from soil in China

**DOI:** 10.3389/fmicb.2025.1705425

**Published:** 2025-10-29

**Authors:** Tongyi Liu, Wei Chen, Ke Zhang, Xiangyu Hu, Alexander Berestetskiy, Qiongbo Hu, Qunfang Weng

**Affiliations:** 1 College of Plant Protection, State Key Laboratory of Green Pesticides, South China Agricultural University, Guangzhou, China; 2 Ganzhou Polytechnic, Ganzhou, Jiangxi, China; 3 All-Russian Research Institute of Plant Protection, Saint Petersburg, Russia

**Keywords:** entomopathogenic fungi, new species, *Gongronella*, *Yunnania*, phylogenetics

## Abstract

Entomopathogenic fungi play a crucial role in integrated pest management by targeting pests through specific infection mechanisms, offering both environmental compatibility and sustainability. In recent years, the growing challenge of pesticide resistance and the increasing demand for green agriculture have made the exploration of novel entomopathogenic fungal resources a major research focus in biological control. In this study, we employed a combination of traditional morphological characterization and multi-locus phylogenetic analyses to identify and describe three new species: *Gongronella yichunensis*, *Gongronella shangraoensis*, and *Yunnania jiujiangensis*. For the genus *Gongronella*, the internal transcribed spacer (ITS) and rRNA large subunit (LSU) regions were used for phylogenetic reconstruction, while the genus *Yunnania* was analyzed using ITS, LSU, β-tubulin (β-TUB), and translation elongation factor (TEF) gene sequences. Furthermore, preliminary bioactivity assessments revealed varying levels of pathogenicity of the new species against *Ostrinia furnacalis*. *Gongronella yichunensis* (strain JX09A02) demonstrated the highest virulence, with a median lethal time (LT_50_) of 7.2 days and a median lethal concentration (LC_50_) of 28.0 × 10^6^ spores/mL. *Gongronella shangraoensis* (strain JX20B02) exhibited intermediate efficacy, showing an LT_50_ of 8.4 days and an LC_50_ of 92.3 × 10^6^ spores/mL. *Yunnania jiujiangensis* (strain JX11B02) displayed relatively lower virulence, with an LT_50_ of 9.5 days and an LC_50_ of 243.8 × 10^6^ spores/mL. These findings not only enrich the genetic resources of entomopathogenic fungi in China but also provide a valuable theoretical and germplasm foundation for developing novel biocontrol agents.

## 1 Introduction

Insect pests pose a severe threat to global agricultural production. Within integrated pest management (IPM) systems, chemical pesticides have long played a dominant role; however, their application has become increasingly limited due to growing resistance, reduced efficacy, and adverse effects on the environment and non-target organisms (Baker et al., 2020; Galli et al., 2024). Currently, global pesticide research and development are shifting significantly toward environmentally friendly biopesticides (Galli et al., 2024). Among these, entomopathogenic fungi, as important microbial insecticides, demonstrate broad application prospects in biological control (Nunes et al., 2024; Shehzad et al., 2025). To date, there are more than 1,000 species of EPF discovered in the world (Gielen et al., 2024). The highly bioactive entomopathogenic fungi (EPF), such as *Beauveria bassiana*, *Metarhizium anisopliae*, and *Purpureocillium lilacinum*, have been widely employed as biological control agents in agricultural pest management and are registered in many countries as mycopesticides (Jiang and Wang, 2023).

Entomopathogenic fungi (EPF) present a sustainable alternative to chemical pesticides due to their lower environmental toxicity, higher biodegradability, and stronger host specificity. Unlike chemicals, EPF typically infects insects by penetrating the cuticle via specialized structures, including appressoria and germ tubes. This contact-based infection mechanism, which is functionally analogous to the “contact toxicity” of chemical insecticides, enables EPF to effectively control piercing-sucking pests such as aphids and whiteflies (Ranadev et al., 2025; Saminathan et al., 2025). This is a characteristic that bacterial and viral insecticides do not possess (Jiang and Wang, 2023). In fact, EPF are not merely entomopathogens killing insects; they play important roles in natural ecosystems through multi-level trophic interactions, such as saprophytes, plant endophytes, and parasites (Vega, 2018; Ranesi et al., 2024). For example, the wheat endophytic *Bea. bassiana* and *Met. robertsii* alters the plants’ secondary metabolites, thereby inhibiting the population growth of insect pests *Rhopalosiphum padi* and *Aphis fabae* (Rasool et al., 2021), while the interactions of *Syncephalastrum* sp. (Mucorales) with fungus gardens and leaf-cutter ants offer new opportunities in their integrated pest management (Barcoto et al., 2017; Bautz et al., 2023). However, EPF still faces challenges such as insufficient resources and suboptimal efficacy, which hinder its development (Deka et al., 2021).

In the pursuit of expanding the repertoire of microbial resources applicable to pest management, this study identified and characterized two novel species of the genus *Gongronella*—*Gongronella yichunensis* and *Gongronella shangraoensis*—along with one new species of the genus *Yunnania*, *Yunnania jiujiangensis*, employing an integrated taxonomic approach combining classical morphological observation with molecular phylogenetic analysis. Moreover, the insecticidal efficacy of these three novel species was evaluated against *Ostrinia furnacalis*. These findings significantly broaden the scope of known microbial diversity and offer valuable germplasm resources for advancing eco-friendly pest control strategies, thereby providing both a theoretical framework and experimental evidence for developing novel bio-insecticides.

## 2 Materials and methods

### 2.1 Soil sample collection

Soil samples were collected in Jiangxi Province, China, in June 2023 using a five-point sampling method. At each sampling site, the topsoil layer (10–20 cm depth) was collected. Each sample, with a weight of approximately 100 g, was placed in a self-sealing bag. The longitude, latitude, specific geographical location, and habitat type of each sampling site were documented in detail (Supplementary Table 1). All samples were stored at 4 °C for subsequent processing (Mariappan et al., 2025).

### 2.2 Fungal isolation and culture

Soil samples were sieved through a 0.45mm mesh to collect the fine soil fraction. Two 10 g aliquots of the fine soil were each mixed with 100 mL of 0.1% Tween-80 solution. The mixtures were vortexed and subsequently allowed to stand for 10 min to form a soil suspension for subsequent use. A 100μL aliquot of the suspension was spread evenly onto a selective medium (containing, per liter of distilled water: 200 g potato, 20 g glucose, 20 g agar, 50 mg chloramphenicol, 50 mg cycloheximide, and 50 mg rose bengal), with three replicates per treatment. The inoculated plates were incubated at 26 ± 1 °C until single colonies were observed. Thereafter, individual colonies were picked with an inoculation loop and transferred to Potato Dextrose Agar (PDA) medium (containing, per liter of distilled water: 200 g potato, 20 g glucose, and 20 g agar) to obtain pure cultures. The purification procedure was repeated iteratively until complete strain purification was achieved (Chen et al., 2021).

### 2.3 Morphological observation of fungal strains

In this study, hyphal and conidial morphology were examined using both light microscopy (MC-D500U, Phoenix, Jiangxi, China) and scanning electron microscopy (SEM). Colony morphology on the obverse and reverse sides was systematically documented on various culture media, including Potato Dextrose Agar (PDA), Dichloran Glycerol (18%) Agar (DG18), Czapek Yeast Extract Agar (CYA), Malt Extract Agar (MEA), and Czapek-Dox Agar (CDA). For SEM sample preparation, fungal plugs (5 mm in diameter) were inoculated onto PDA plates alongside sterile aluminum foil strips. The cultures were incubated in darkness at 26 ± 1 °C until approximately two-thirds of the foil surface was colonized by mycelia. The foil specimens with adherent mycelia and spores were then subjected to fixation, dehydration, critical-point drying, and gold sputtering prior to SEM imaging (Chen et al., 2024).

### 2.4 DNA extraction, PCR amplification, and sequencing

Fungal genomic DNA was extracted from colonies grown on PDA for a week, using a fungal DNA extraction kit (OMEGA, D3195-02) and the protocol provided by the manufacturer. The genomic loci: internal transcribed spacer (ITS), rRNA large subunit (LSU), translation elongation factor 1-alpha (TEF), β-tubulin (β-tub) were amplified by PCR. For PCR, the primer pairs, ITS1/ITS4, LR5/LR0R, EF983F/EF2218R, and BT2a/BT2b (Castlebury et al., 2004; Rehner et al., 2011), were employed. The PCR products were validated by 1% agarose gel electrophoresis to display a single clear band, and then they were sequenced by the Sanger method (performed by Zhejiang Youkang Biotechnology Co., Ltd., Hangzhou, China).

### 2.5 Molecular phylogenetic analysis

By incorporating representative strains of the target genus from the GenBank database, a concatenated multi-locus phylogenetic analysis was performed using appropriate genetic markers to further elucidate the taxonomic placement of these isolates. Phylogenetic analyses were carried out using both Maximum Likelihood (ML) and Bayesian Inference (BI) methods. Sequence alignment was performed using the MAFFT version 7 web server,^1^ employing the iterative optimization strategy (FFT-NS-i) for multiple sequence alignment (MSA) (Nakamura et al., 2018). The low-quality alignment positions of sequences were removed by Gblocks (Xiang et al., 2023). The optimal partitioning strategy and evolutionary models for phylogenetic tree construction were selected using ModelFinder for both IQ-TREE and MrBayes (Kalyaanamoorthy et al., 2017). An ML phylogenetic tree was constructed using IQ-TREE software. To assess the stability of the tree topology, bootstrap analysis was conducted with 1,000 repetitions. A BI phylogenetic tree was constructed using MrBayes software. To ensure reliable results, the Markov Chain Monte Carlo (MCMC) run was set to 2,000,000 generations, with sampling every 1,000 generations, and the first 25% of the unstable samples were discarded (Ronquist et al., 2012). The phylogenetic trees generated by both ML and BI analyses were visualized using MEGA v7 and FigTree V 1.4.3. The sequence information for the strains investigated in this study is summarized in Supplementary Tables 2, 3.

### 2.6 Bioactivity assays of fungal strains

The experimental subjects were third-instar larvae of *Ostrinia furnacalis*, which had been laboratory-reared for five generations with an artificial diet (43 g cornmeal, 43 g soybean flour, 26 g yeast extract, 26 g glucose, 1.5 g multivitamin complex, 5.7 g agar, and 1.5 g sorbic acid). Bioassays were conducted following the China Agricultural Industry Standard NY/T 1154.6–2006 for insecticidal bioassays in the laboratory, with minor modifications (Chen et al., 2024). Conidia of the tested strains were suspended in a 0.05% Tween 80 solution to a stock of 1 × 10^8^, 1 × 10^7^, 1 × 10^6^, and 1 × 10^5^ spores/mL. The third-instar larvae of *O. furnacalis* were immersed in conidia suspension for 10 s. After dryness, the larvae were transferred to individual dishes and incubated at 25 ± 1 °C. Larvae were fed the artificial diet, which was changed every 2 days. Mortalities were recorded every day, and the dead larvae were transferred to Petri dishes for incubation for mycelial growth. Subsequently, the fungi emerging from the cadavers were re-isolated onto PDA plates and identified morphologically to confirm pathogenicity in accordance with Koch’s postulates. Control groups were treated with a 0.05% Tween-80 solution. There were three repeats with 10 larvae in each treatment. The same test was replicated twice. An ANOVA was performed using SPSS version 26.0 (IBM, USA) to test the significance. Data were compared with Duncan’s multiple range test (DMRT). Statistical significance was considered at a *p*-value of < 0.05. The values of LT_50_ and LC_50_ were evaluated based on the probit regression analysis (LC-p/LT-p analysis). The DPS version 9.01 (Data Processing System, Hongzhou, China) software was used to complete the statistical analyses.

## 3 Results

### 3.1 Phylogenetic analysis reveals new species

#### 3.1.1 Genus *Gongronella*

**Figure 1:**
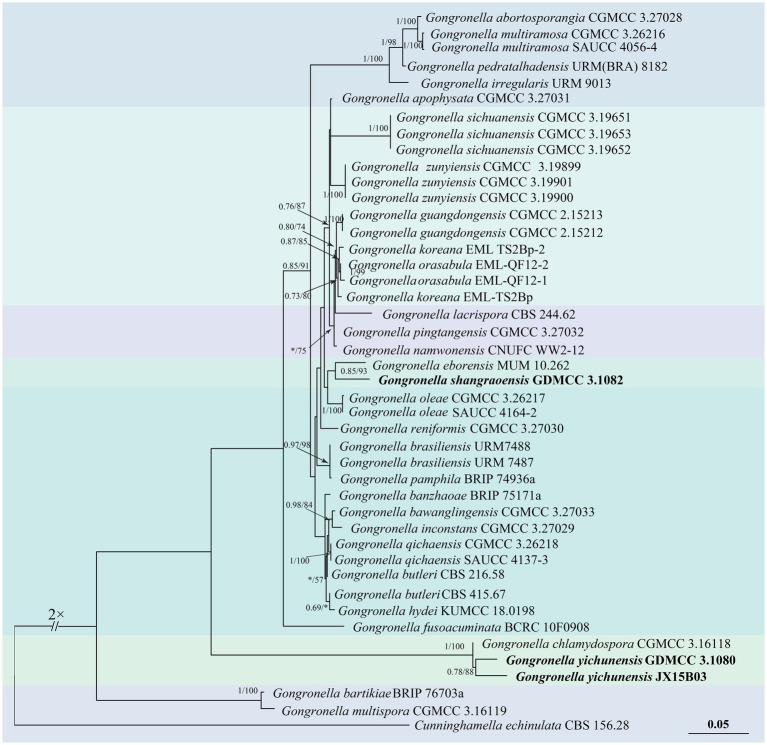
Phylogenetic tree of *Gongronella* generated from a concatenated multi-locus dataset. The tree was constructed based on the sequences of ITS and LSU. Bayesian posterior probability (BI ≥ 0.50) and IQtree bootstrap support values (ML ≥ 50) are labeled on the branch in the following order: BI/ML. BI < 0.50 and ML < 50 are marked as “*,” which suggests the branch with lower credibility. *Cunninghamella echinulata* (CBS 156.28) served as the outgroup of the phylogenetic trees, while the other type strains were retrieved from the MycoBank database (https://www.mycobank.org/).

Based on the ITS and LSU sequences, a phylogenetic tree of the genus *Gongronella* was constructed using the putative new species strains JX20B02 (GDMCC 3.1082), JX09A02 (GDMCC 3.1080), and JX15B03, as well as the typical material of the species retrieved from the NCBI database.^2^ *Cunninghamella echinulata* (CBS 156.28) was used as the outgroup. The analysis was performed using the Bayesian method (GTR + F + G4 model) and IQtree analysis (TN + F + R2 model). The multi-gene dataset consisted of 1,465 base pairs, derived from the ITS (1–533 bp) and LSU (534–1,465 bp) regions. The results indicated that the strain JX20B02 (GDMCC 3.1082) with *G. eborensis* forms a well-supported clade with the Bayesian posterior probability (BI) and IQtree bootstrap support (BI = 0.85, ML = 93). Meanwhile, the strains JX09A02 (GDMCC 3.1080) and JX15B03 formed a clade (BI = 0.78, ML = 88) and clustered together with *G. chlamydospora* (BI = 1, ML = 100) (Figure 1).

#### 3.1.2 Genus *Yunnania*

**Figure 2:**
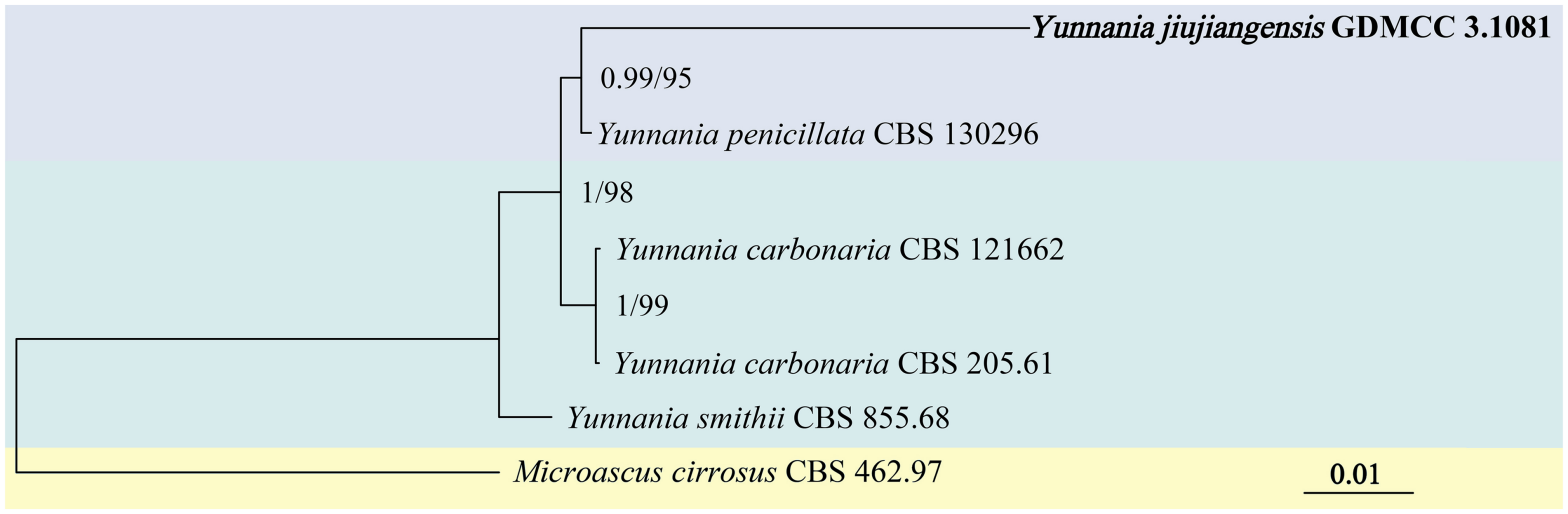
Phylogenetic tree of *Yunnania* generated from a concatenated multi-locus dataset. The tree was constructed based on the sequences of ITS, LSU, TEF, and β-tub. The concatenated dataset comprised 2,879 bp, including ITS (1–585 bp), LSU (586–1,454 bp), TEF-*α* (1455–2,378 bp), and tub2 (2379–2,879 bp). Bayesian posterior probabilities (BI ≥ 0.50) and IQtree bootstrap support values (ML ≥ 50) were annotated on branches in the order of BI/ML. *Microascus cirrosus* CBS 462.97 was designated as the outgroup for the phylogenetic tree.

Phylogenetic analysis of the genus *Yunnania* was conducted using a concatenated dataset of four loci from six taxa, with *Microascus cirrosus* CBS 462.97 as the outgroup. The concatenated dataset comprised 2,879 bp, including ITS (1–585 bp), LSU (586–1,454 bp), TEF (1,455–2,378 bp), and β-tub (2379–2,879 bp). The analysis was performed using the Bayesian method (GTR + G + F model) and IQtree analysis (TN + G4 + F model). The results showed that JX11B02 (GDMCC 3.1081) and *Y. penicillata* clustered within a major clade (BI/ML = 0.99/95) but exhibited considerable genetic distance between them (Figure 2).

### 3.2 Taxonomy

*Gongronella yichunensis* Q. Hu & W. Chen, sp. nov.

Mycobank number: MB859592. Index Fungorum number: IF903766.

Etymology: Named after the type locality, Yichun City (Jiangxi Province, China).

**Figure 3:**
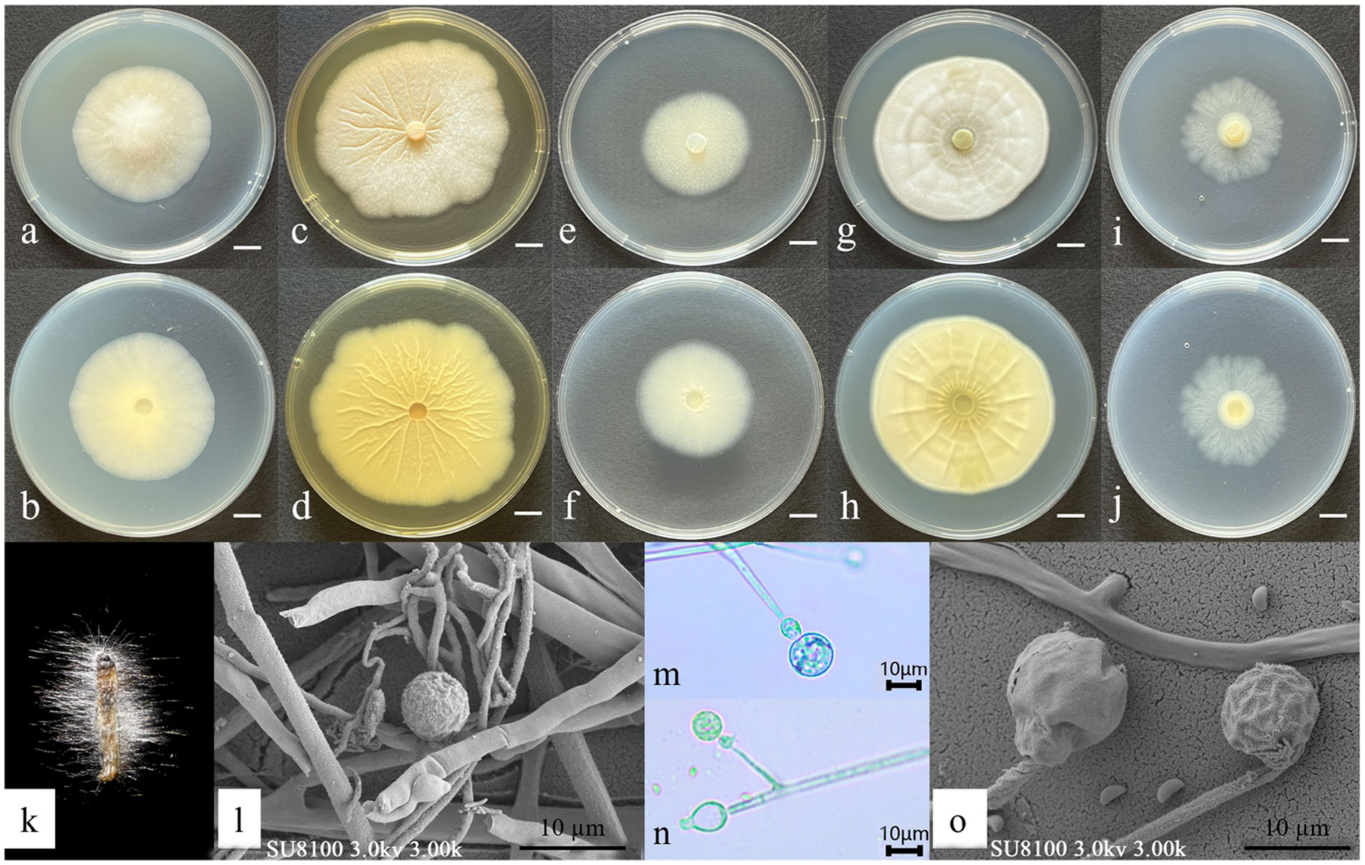
Morphological characteristics of *Gongronella yichunensis.* **(a,c,e,g,i)** Colony obverse at 7-day cultivation on PDA/MEA/DG18/CYA/CDA plate; **(b,d,f,h,j)** colony reverse at 7-day cultivation on PDA/MEA/DG18/CYA/CDA plate; **(k)** symptom of *O. furnacalis* treated the fungus after 4 days; **(l,m,n)** sporangiophores with variously shaped apophyses and sporangia; **(o)** sporangia and sporangiospores. — Scale **(a–j)** = 1 cm.

Asexual morph: Hyphae hyaline, 5.10–7.28 μm wide. Sporangiophores, borne on aerial hyphae, erect to slightly curved, sympodially branched, hyaline, smooth-walled, 17.8–70.8 × 1.6–3.23 μm, and aseptate or with one septum. Fertile sporangia, hyaline, globose, 10.14–12.14 μm in diameter, and smooth-walled. Apophyses, hyaline, smooth-walled, predominantly ellipsoid, 6.18–7.27 × 5.36–5.91 μm. Sporangiospores, hyaline, smooth-walled, mainly reniform, and 2.35–3.52 × 1.17–1.41 μm in diameter. Rhizoids, hyaline, branched, and irregularly shaped (Figure 3). Chlamydospores and Zygospores: not observed.

Culture characteristics (after 7 days of incubation at 26 ± 1 °C in darkness): PDA: 47.33 ± 0.47 mm diameter, obverse creamy white, cottony texture; reverse pale yellow. CDA: 36.67 ± 0.94 mm diameter, obverse irregular, circular form with a white, cottony texture; reverse white. CYA: 57.67 ± 0.47 mm, obverse white, velvety texture with distinctive concentric ring patterns and radiating fissures; reverse pale yellow with prominent radiating solar-like patterns. DG18: 37.33 ± 0.94 mm, obverse creamy texture; reverse milky white. MEA: 63.67 ± 0.47 mm, obverse irregular, felt-like with clear radiating fissures; reverse pale yellow (Figure 3).

Holotype: Strain JX09A02 (GDMCC 3.1080), isolated from crop soil in Yifeng County, Yichun City, Jiangxi Province, China. Collected by W. Chen in June 2023. The strain is preserved in the Guangdong Microbial Culture Collection Center (GDMCC).

Note: *G. yichunensis* and *G. chlamydospora* clustered within a single clade, indicating a close phylogenetic relationship between the two species. However, they exhibit significant morphological differences compared to *G. chlamydospora*. The two species can be distinguished morphologically by their sporangium diameter, *G. yichunensis* with a significantly smaller sporangium diameter (ca. 11.14 μm) compared to *G. chlamydospora* (19.5 μm). Furthermore, sequence comparison of the ribosomal ITS regions revealed 20 bp differences between *G. yichunensis* and *G. chlamydospora*.

*Gongronella shangraoensis* Q. Weng & W. Chen, sp. nov.

Mycobank number: MB859593. Index Fungorum number: IF903767.

Etymology: Named after the type locality, Shangrao City (Jiangxi Province, China).

**Figure 4:**
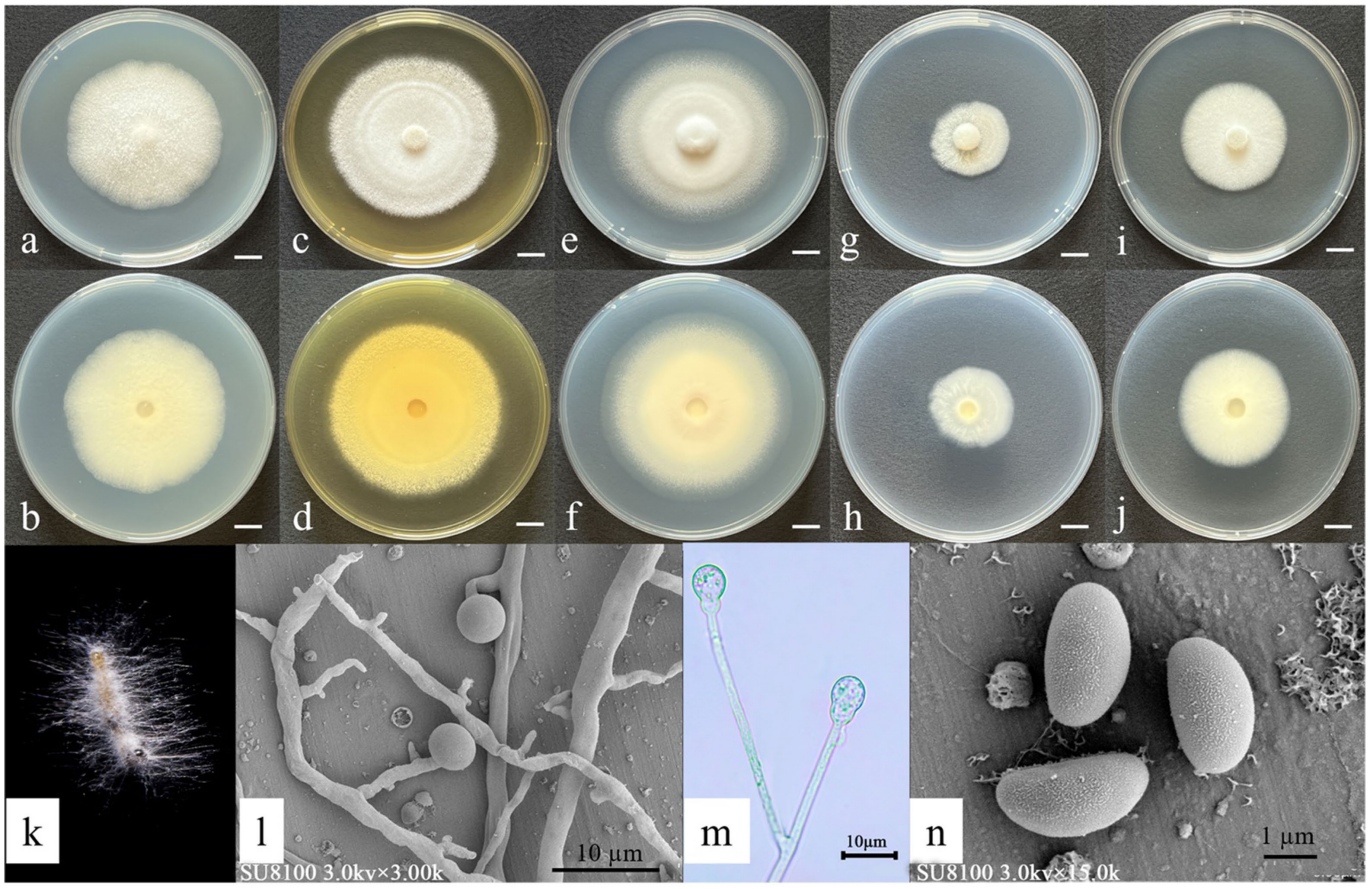
Morphological characteristics of *Gongronella shangraoensis.* **(a,c,e,g,i)** Colony obverse at 7-day cultivation on PDA/MEA/CYA/CDA/DG18 plate; **(b,d,f,h,j)** colony reverse at 7-day cultivation on PDA/MEA/DG18/CYA/CDA plate; **(k)** symptom of *O. furnacalis* treated the fungus after 4 days; **(l,m)** sporangiophores with variously shaped apophyses and sporangia; **(n)** Sporangiospores. — Scale **(a–j)** = 1 cm.

Asexual morph: Hyphae hyaline: 3.82–5.76 μm wide. Sporangiophores: borne on aerial hyphae, erect, sympodially branched, 21.2–42.5 × 1.49–2.25 μm, hyaline, smooth-walled, and mostly aseptate or with one septum. Fertile sporangia: hyaline, terminal on sporangiophores, globose, 4.14–4.86 μm in diameter, and smooth-walled. Apophyses: hyaline, smooth-walled, and globose to ovoid (2.06–2.38 × 2.30–3.71 μm). Sporangiospores: hyaline, smooth-walled, predominantly ovoid to reniform, and 2.60–2.87 × 1.40–1.53 μm. Rhizoids: hyaline, branched, and irregularly shaped (Figure 4). Chlamydospores and Zygospores: not observed.

Culture characteristics (after 7 days of incubation at 26 ± 1 °C in darkness): PDA: diameter of 51.33 ± 1.25 mm; obverse off-white, cottony texture; reverse pale yellow. CDA: 37 ± 1.63 mm; obverse irregular circular shapes, cottony white texture; reverse white. CYA: 63 ± 0.82 mm; obverse floccose white surface with concentric rings and radial cracks; reverse pale yellow with distinct radial striations. DG18: 38 ± 0.82 mm; obverse cream-colored; reverse off-white. MEA: 65.33 ± 3.77 mm; obverse irregular, felt-like, with distinct radial cracks; reverse pale yellow (Figure 4).

Holotype: Strain JX20B02 (GDMCC 3.1082) was isolated from forest soil in Guangfeng District, Shangrao City, Jiangxi Province, China, collected by Wei Chen in June 2023. The strain is preserved in the Guangdong Microbial Culture Collection Center (GDMCC).

Note: *G. shangraoensis* and *G. eborensis* demonstrated a close relationship; however, they can be distinguished morphologically by the size of their sporangia. *G. shangraoensis* exhibits a significantly smaller sporangium diameter (ca. 4.50 μm) compared to *G. eborensis* (11.75 μm). Furthermore, sequence comparison of the ribosomal ITS and LSU regions revealed 17 bp and 11 bp differences between *G. shangraoensis* and *G. eborensis* in the ITS and LSU regions, respectively.

*Yunnania jiujiangensis* W. Chen & Q. Weng, sp. nov.

Mycobank number: MB859596. Index Fungorum number: IF903765.

Etymology: Named based on the isolated location Jiujiang, Jiangxi Province.

**Figure 5:**
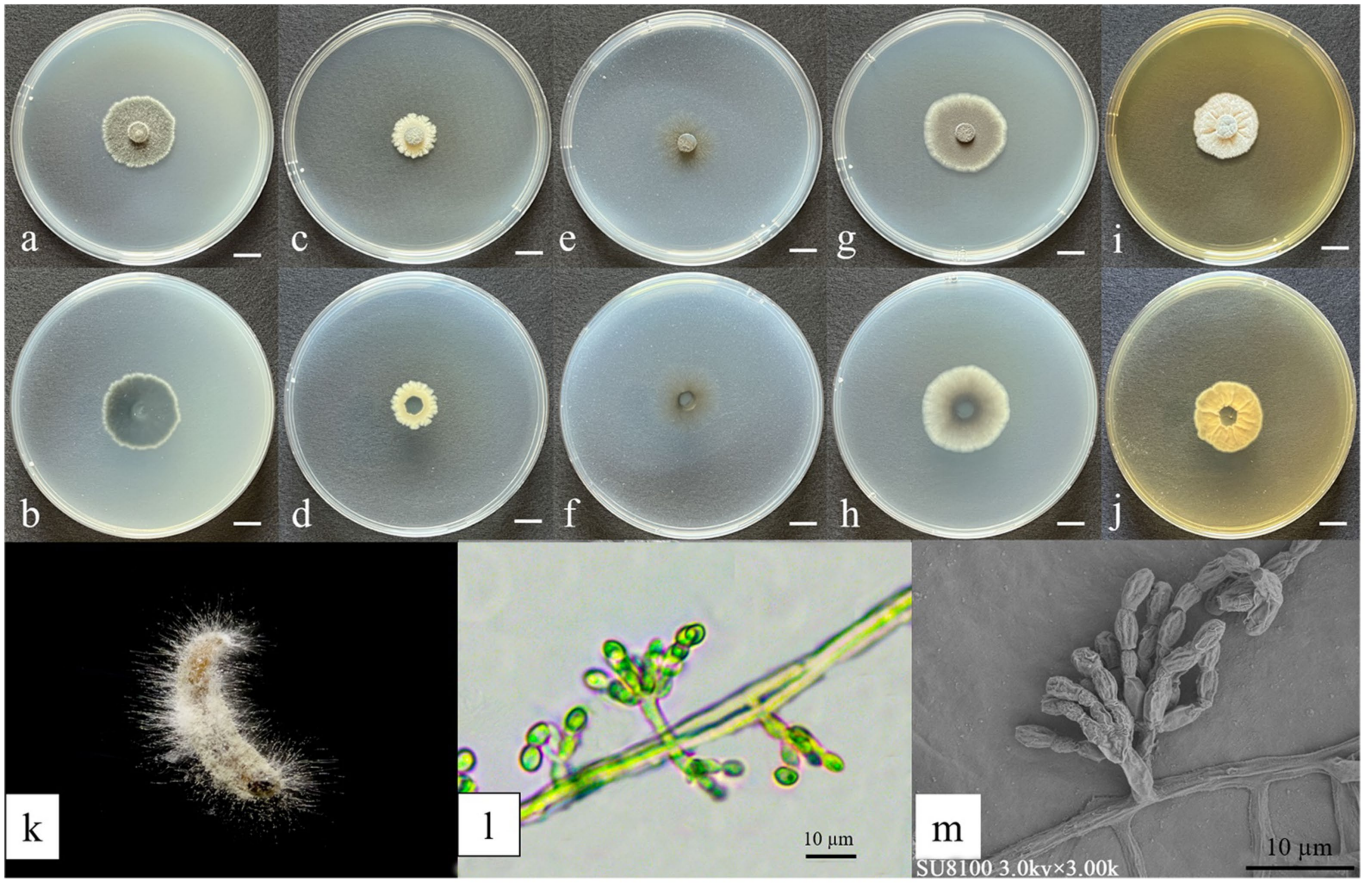
Morphological characteristics of *Yunnania jiujiangensis*. **(a,c,e,g,i)** Colony obverse at 7-day cultivation on PDA/DG18/CDA/CYA/MEA plate; **(b,d,f,h,j)** colony reverse at 7-day cultivation on PDA/MEA/DG18/CYA/CDA plate; **(k)** symptom of *O. furnacalis* treated the fungus after 4 days; **(l)** Sporulating structures and conidia; **(m)** Conidia. — Scale **(a–j)** = 1 cm.

Asexual morph: Vegetative hyphae: hyaline, 2.21–3.08 μm wide. Conidiophores: solitary, hyaline, erect, arising from aerial hyphae, mycelium-like, without a distinct stipe, 4.66–9.32 × 2.21–3.43 μm, and smooth-walled. Phialides: borne directly on the conidiophores, monoverticillate, and 5.24–8.16 × 0.36–1.74 μm. Conidia: ellipsoidal, smooth-walled, truncate at base, 4.37–6.31 × 3.40–3.78 μm, and forming irregular to regular cylindrical chains (Figure 5).

Cultural characteristics (after 7 days of incubation at 26 ± 1 °C in darkness): PDA: 22.33 ± 0.47 mm; obverse jet-black center with white cottony margin; reverse black centrally, white peripherally. DG18: 15.67 ± 0.47 mm; obverse pale yellow with irregularly serrate margin; reverse light yellow peripherally, dark green centrally. CDA: 18.92 ± 0.82 mm; obverse olive-brown, floccose with radiating margin; reverse uniformly olive-brown. CYA: 28.67 ± 0.47 mm; obverse regularly circular, dark green velvety texture, center with white margin; reverse dark green centrally fading to white. MEA: 23.67 ± 1.25 mm; grayish-white with distinct radial striations; reverse yellowish-brown peripherally, dark green centrally (Figure 5).

Holotype: Strain JX11B02 (GDMCC 3.1081) was isolated from soil in a forest habitat in Pengze County, Jiujiang City, Jiangxi Province, China. The sample was collected by Wei Chen in June 2023. The strain is preserved in the Guangdong Microbial Culture Collection Center (GDMCC).

Notes: Phylogenetic analysis showed that strains *Y. jiujiangensis* and *Y. penicillata* clustered in one clade with a close relationship. *Y. jiujiangensis* is similar to *Y. penicillata*, but the former phialides are borne directly on the conidiophores, metulae are rarely present or absent, penicilli exhibit fewer tiers of branching, typically monoverticillate; while the latter metulae are conspicuously present, phialides arise from metulae, and penicilli display more complex branching with two or more tiers. Furthermore, the conidial dimensions of the former (4.37–6.31 × 3.40–3.78 μm) are notably larger than those of the latter (4.0–5.9 × 2.5–3.5 μm).

### 3.3 Bioactivity of the new species against *Ostrinia furnacalis*

**Figure 6:**
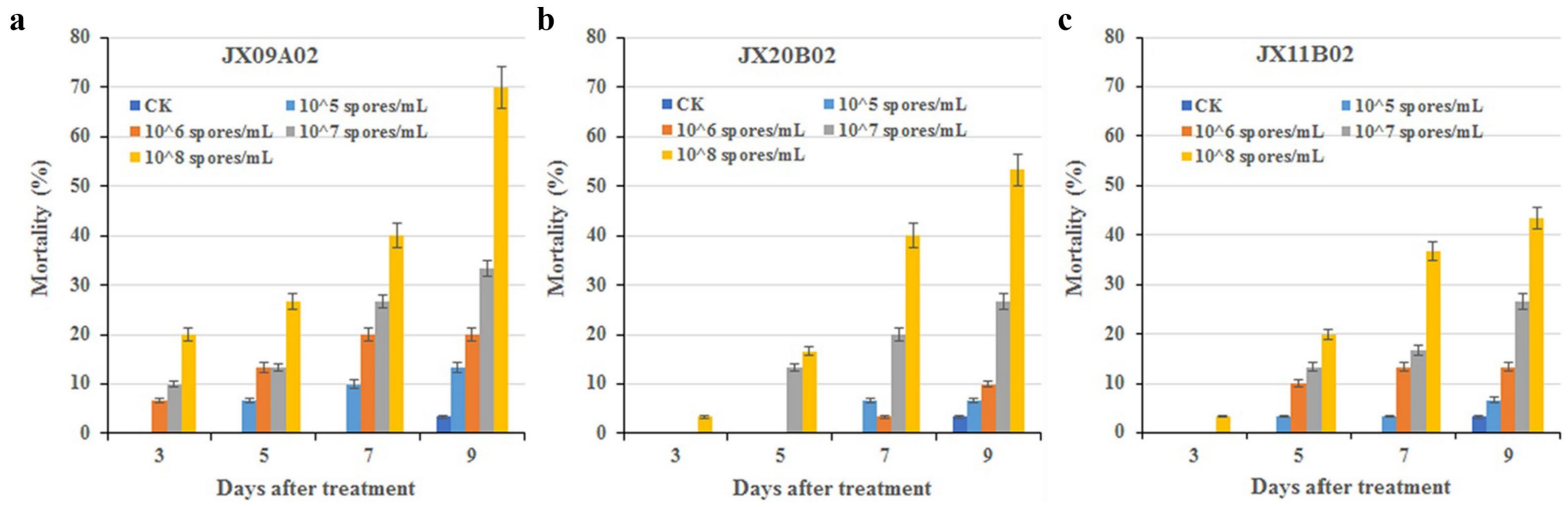
Bioassay of new species against 3rd instar larvae of *Ostrinia furnacalis.* **(a)** *G. yichunensis* JX09A02 (GDMCC 3.1080). **(b)** *G. shangraoensis* JX20B02 (GDMCC 3.1082). (C) *Y. jiujiangensis* JX11B02 (GDMCC 3.1081). Control groups were treated with a 0.05% Tween-80 solution.

All three fungal strains exhibited certain levels of pathogenicity against *Ostrinia furnacalis*. On day 9 post-treatment, insect mortality rates in groups treated with a suspension concentration of 1 × 10^8^ spores/mL exceeded 40%. Specifically, strain JX09A02 induced a mortality rate of 70%, while JX20B02 reached 53.33%. Strain JX09A02 demonstrated the highest virulence, with a median lethal time (LT_50_) of 7.2 days and a median lethal concentration (LC_50_) of 28.0 × 10^6^ spores/mL. Strain JX20B02 showed intermediate efficacy, with an LT_50_ of 8.4 days and an LC_50_ of 92.3 × 10^6^ spores/mL. Strain JX11B02 displayed relatively lower virulence, requiring 9.5 days for LT_50_ and achieving LC_50_ at 243.8 × 10^6^ spores/mL. The relevant parameters are provided in Table 1 and Figure 6.

**Table 1:** Estimation of LC_50_/LT_50_ of new fungal species against third instar larvae of *Ostrinia furnacalis.*

LT-p analysis (1 × 10^8^ spores/mL treatment)								
Strain	Intercept	Slope	SE	Correlation Coefficient	χ^2^	DF	p*	LT_50_ and 95% CI (d)
*G. yichunensis* JX09A02	2.6744	2.7103	0.5089	0.9081	6.4637	2	0.0395	7.2 (6.2–9.1)
*Y. jiujiangensis* JX11B02	1.7535	3.3279	0.6157	0.9867	1.1663	2	0.5581	9.5 (7.9–13.3)
*G. shangraoensis* JX20B02	1.2059	4.1052	0.6576	0.9980	0.3004	2	0.8605	8.4 (7.3–10.6)

LC-p analysis (9th day of post-treatment)								
Strain	Intercept	Slope	SE	Correlation coefficient	χ^2^	DF	*p*	LC_50_ and 95% CI (10^6^ spores/mL)
*G. yichunensis* JX09A02	0.3913	0.6188	0.1004	0.9649	3.2805	2	0.1939	28.0 (12.1–105.8)
*Y. jiujiangensis* JX11B02	0.5853	0.5264	0.1171	0.9994	0.0365	2	0.9819	243.8 (52.2–8741.4)
*G. shangraoensis* JX20B02	−0.6564	0.7101	0.1373	0.9870	0.4408	2	0.8022	92.3 (30.2–872.2)

*When the *p*-value is ≥ 0.05, it means that there is no significant inconsistency between the data and the model, so the result is more reliable.

## 4 Discussion

EPF are often soil inhabitants; large numbers of EPF can persist for a long time in soil. Sometimes, they can also reproduce in the rhizosphere of plants, thereby maintaining a certain population level in the soil (Chen et al., 2021). Following infection by entomopathogenic fungi (EPF), the fungus proliferates extensively on or within the insect host, producing numerous spores (Ma et al., 2024). These spores are then released into the environment. A portion of these dispersed spores can enter a dormant state in the soil, which enhances their survival during unfavorable conditions like cold or drought. This dormancy allows them to persist until the return of suitable hosts or growth conditions triggers their germination (Shin et al., 2020; Quesada-Moraga et al., 2024). Furthermore, soil is a key source for EPF genetic resources. In recent years, many new EPF species have been isolated and identified from soil (Konopická et al., 2024; Sun et al., 2024; Liu et al., 2025). In practice, we identified several factors that influence the EPF isolation. First isolating fungi from excessively moist soils is challenging because high humidity severely affects fungal survival in the soil (Romero et al., 2022). Second, the storage conditions of soil samples are critical. Typically, storing soil samples at 0 °C for 3–6 months does not affect fungal isolation from the soil; however, prolonged storage leads to a rapid decrease in the number of fungi present in the samples. Furthermore, repetitive operations also affect the isolation results of fungal species in each sample, and repeating the process twice can significantly increase the number of isolated fungal species. Variations in collection sites and sampling times may also contribute to discrepancies in the results (Wang et al., 2025). This study has effectively controlled the aforementioned factors; therefore, the research findings presented are credible and reliable.

The phylogenetic analysis methods in fungal taxonomic research have undergone a critical paradigm shift from single-locus to multilocus approaches. Although early studies widely relied on single loci such as ITS for preliminary identification due to their accessibility and comprehensive database coverage, these markers exhibit limited phylogenetic information and low resolution, often failing to accurately resolve interspecific or intraspecific relationships among recently diverged or complex lineages (Luecking et al., 2020; Kauserud, 2023). Moreover, single-gene analyses are prone to topological errors caused by gene introgression or incomplete lineage sorting. With advances in molecular techniques, multilocus phylogenetic analysis has become the standard practice in contemporary taxonomic studies (Dong et al., 2024). By integrating multiple genetic markers—including ribosomal regions (ITS, LSU) and protein-coding genes (e.g., TEF1-*α*, β-tubulin, RPB1, and RPB2)—this approach significantly enhances nodal support and robustness in phylogenetic inference (Heled and Drummond, 2010; Manawasinghe et al., 2025). Consequently, it not only improves the resolution of morphologically similar or cryptic species but also provides a reliable evolutionary framework for reassessing higher taxonomic ranks. Nie et al. conducted a multi-locus phylogenetic analysis employing sequences of the large subunit nuclear ribosomal DNA (nLSU), small subunit nuclear ribosomal DNA (nSSU), mitochondrial small subunit ribosomal DNA (mtSSU), and translation elongation factor 1-alpha (tef1). This comprehensive study necessitated a taxonomic revision of the classical circumscription of Conidiobolus, resulting in the proposal of three novel genera: *Capillidium*, *Microconidiobolus*, and *Neoconidiobolus* (Nie et al., 2020). Kobmoo et al. employed an integrated research strategy combining phylogenetic analysis, morphological observation, liquid chromatography–mass spectrometry (LC–MS)-based metabolomics, and virulence assays to systematically resolve taxonomic issues in the *Metarhizium anisopliae* species complex (Kobmoo et al., 2024). This study provides comprehensive theoretical guidance for addressing challenges in delineating closely related species and infraspecific taxonomic units. Such an integrated approach, based on cross-disciplinary and cross-institutional collaboration, can significantly benefit future fungal taxonomic studies.

In this study, species identification was accomplished through an integrated taxonomic strategy combining morphological observations with molecular phylogenetic analyses (Wang et al., 2024; Lin et al., 2025). To strengthen phylogenetic robustness, multi-locus phylogenetic reconstructions were performed using the internal transcribed spacer (ITS), large subunit of nuclear ribosomal RNA (nrLSU), translation elongation factor 1-alpha (tef1), and beta-tubulin (tub2) gene regions. Isolates were cultivated on diverse culture media to systematically record phenotypic and developmental characteristics. Ultimately, three novel species were delineated: *Gongronella yichunensis*, *Gongronella shangraoensis*, and *Yunnania jiujiangensis*.

This study conducted a preliminary assessment of the bioactivity of the newly identified fungal species. The results demonstrated that strains JX09A02 (*G. yichunensis*), JX11B02 (*Y. jiujiangensis*), and JX20B02 (*G. shangraoensis*) all exhibited varying levels of insecticidal activity against the lepidopteran pest *Ostrinia furnacalis*, highlighting their potential as biocontrol agents. However, these findings, derived from initial experiments with a single target pest, are insufficient to fully evaluate their practical efficacy. To bridge this knowledge gap, a two-pronged approach is recommended for future research. First, subsequent laboratory studies should expand the range of target pests to include other taxonomically important groups (e.g., Coleoptera and Hemiptera) to systematically elucidate their insecticidal spectrum and host range. Second, and crucially, laboratory-based bioassays cannot replicate the complex field environment. Therefore, systematic field trials are urgently needed to translate these findings into practical pest management strategies. In such trials, to maximize spore viability and infection efficiency, the application of fungal inoculants should be preferentially conducted during periods such as late afternoon, on overcast days, or when crop canopy humidity remains high, thereby mitigating the detrimental effects of UV irradiation and facilitating spore germination and host invasion (Jamunarani et al., 2022).

Furthermore, the mechanisms underlying their bioactivity require in-depth investigation. It is hypothesized that the activity may involve one or more modes of action, including cuticular penetration, toxin secretion, immunosuppression, or nutrient competition. These mechanisms should be validated through multidisciplinary approaches such as histopathology, molecular toxicology, and metabolomics. Elucidating the mode of action will not only enhance the understanding of the physiological and ecological interactions between the fungal strains and their hosts but also provide a theoretical foundation for the targeted selection of highly active strains, the development of effective formulations, and the design of field application strategies.

## 5 Conclusion

Through a combined multigene phylogenetic analysis and morphological examination, this study successfully identified and described three novel species: *Gongronella yichunensis*, *Gongronella shangraoensis*, and *Yunnania jiujiangensis*. Preliminary insecticidal activity assessment revealed significant biocontrol potential of these species against larvae of *Ostrinia furnacalis*, demonstrating their practical value in pest management. These findings not only expand the repository of entomopathogenic fungal resources in China but also provide a crucial theoretical and germplasm foundation for the development of novel biocontrol agents.

## Data Availability

The datasets presented in this study can be found in online repositories. The names of the repository/repositories and accession number(s) can be found in the article/Supplementary material.

## Footnotes

^1^ https://mafft.cbrc.jp/alignment/server/

^2^ https://www.ncbi.nlm.nih.gov/
