# Correction: Three new entomopathogenic fungal species isolated from soil in China

**DOI:** 10.3389/fmicb.2025.1737534

**Published:** 2025-11-18

**Authors:** Tongyi Liu, Wei Chen, Ke Zhang, Xiangyu Hu, Alexander Berestetskiy, Qiongbo Hu, Qunfang Weng

**Affiliations:** 1 College of Plant Protection, State Key Laboratory of Green Pesticides, South China Agricultural University, Guangzhou, China; 2 Ganzhou Polytechnic, Ganzhou, Jiangxi, China; 3 All-Russian Research Institute of Plant Protection, Saint Petersburg, Russia

**Keywords:** entomopathogenic fungi, new species, *Gongronella*, *Yunnania*, phylogenetics

The funder Russian Science Foundation, grant number 24-46-00005 was erroneously omitted.

The original version of this article has been updated.

