# Supplementary material for: Three new entomopathogenic fungal species isolated from soil in China
Source: Front Microbiol. 2025 Oct 29;16:1705425. doi: 10.3389/fmicb.2025.1705425 (PMC12605131; doi:10.3389/fmicb.2025.1705425)
Supplement: Supplementary file 1 [file Data_Sheet_1.docx]

Supplementary Material

Supplementary Table 1 Sample information.

| Sample  Number | Strain | Collection Site | Vegetation | Latitude and  Longitude |
| --- | --- | --- | --- | --- |
| JX09 | JX09A02 | Yichun City, Jiangxi Province, China | Crop | 28.3891°N, 114.7672°E |
| JX11 | JX11B02 | Jiujiang City, Jiangxi Province, China | Forest | 29.7943°N, 116.5754°E |
| JX15 | JX15B03 | Ganzhou City, Jiangxi Province, China | Forest | 26.9168°N, 116.0076°E |
| JX20 | JX20B02 | Shangrao City, Jiangxi Province, China | Forest | 28.3824°N, 118.2482°E |

Supplementary Table 2 Strain information used for phylogenetic analysis of the genus *Gongronella*

| Species | Voucher information | Location | GenBank accession number | |
| --- | --- | --- | --- | --- |
|  |  |  | ITS | LSU |
| *G. banzhaoae* | BRIP 75171a | Australia | OR271908 | OR259049 |
| *G. brasiliensis* | URM 7487 | Brazil | NR155148 | KY114932 |
|  | URM 7488 | Brazil | KY114931 | KY114933 |
| *G. butleri* | CBS 415.67 | Netherlands | JN206288 | MH870714 |
|  | CBS 216.58 | UK | JN206285 | MH869292 |
| *G. chlamydospora* | CGMCC 3.16118 | China | OL678157 | PQ399921 |
| *G. eborensis* | MUM 10.262 | Portugal | KT809408 | MN947301 |
| *G. guangdongensis* | CGMCC 2.15212 | China | NR158464 | MN947303 |
|  | CGMCC 2.15213 | China | KC462740 | MN947304 |
| *G. hydei* | KUMCC 18.0198 | China | NR171964 | MT907273 |
| *G. koreana* | EML-TS2Bp | South Korea | KP636529 | KP636530 |
|  | EML-TS2Bp-2 | South Korea | KP835545 | KP835542 |
| *G. lacrispora* | CBS 244.62 |  | MH858146 | JN206609 |
| *G. multiramosa* | CGMCC 3.26216 | China | OR733546 | OR733611 |
|  | SAUCC 4056-4 | China | OR733545 | OR733610 |
| *G. multispora* | CGMCC 3.16119 | China | OL678158 | PQ399922 |
| *G. namwonensis* | CNUFC WW2-12 | South Korea | NR175640 | MN658482 |
| *G. oleae* | SAUCC 4164-2 | China | OR742079 | OR733609 |
|  | CGMCC 3.26217 | China | OR742078 | OR733608 |
| *G. orasabula* | EML-QF12-1 | South Korea | KT936269 | KT936263 |
|  | EML-QF12-2 | South Korea | KT936270 | KT936264 |
| *G. pamphilae* | BRIP 74936a | Australia | NR189982 | OR259050 |
| *G. qichaensis* | CGMCC 3.26218 | China | OR733544 | OR733607 |
|  | SAUCC 4137-3 | China | OR733543 | OR733606 |
| *G. pedratalhadensis* | URM(BRA) 8182 | Brazil | MN912512 | MN912508 |
| ***G. shangraoensis*** | **GDMCC 3.1082** | **China** | **PP907791** | **PP495838** |
| *G. sichuanensis* | CGMCC 3.19651 | China | MK813373 | MK813855 |
|  | CGMCC 3.19652 | China | MK813374 | MK813856 |
|  | CGMCC 3.19653 | China | MK813375 | MK813857 |
| ***G. yichunensis*** | **GDMCC 3.1080** | **China** | **PP905125** | **PP495827** |
|  | **JX15B03** | **China** | **PV112675** | **PP495828** |
| *G. zunyiensis* | CGMCC 3.19899 | China | MN453856 | MN453853 |
|  | CGMCC 3.19900 | China | MN453857 | MN453854 |
|  | CGMCC 3.19901 | China | MN453858 | MN453855 |
| *G. abortosporangia* | CGMCC 3.27028 | China | PP195847 | PP195948 |
| *G. apophysata* | CGMCC 3.27031 | China | PP195853 | PP195954 |
| *G. bawanglingensis* | CGMCC 3.27033 | China | PP195857 | PP195958 |
| *G. inconstans* | CGMCC 3.27029 | China | PP195849 | PP195950 |
| *G. pingtangensis* | CGMCC 3.27032 | China | PP195855 | PP195956 |
| *G. reniformis* | CGMCC 3.27030 | China | PP195851 | PP195952 |
| *G. bartikiae* | BRIP 76703a | Australia | PQ882522 | PV069357 |
| *G. fusoacuminata* | BCRC 10F0908 | Taiwan | NR_199105 | PQ496505 |
| *G. irregularis* | URM 9013 | Brazil | PP923717 | PP923718 |
| *Cunninghamella echinulata* | CBS 156.28 |  | JN205895 | MH877699 |

Supplementary Table 3 Strain information used for phylogenetic analysis of the genus *Yunnania*

| Species | Voucher information |  | GenBank accession number | | | |
| --- | --- | --- | --- | --- | --- | --- |
|  |  | Location | ITS | *β-tub* | TEF | LSU |
| *Y. carbonaria* | CBS 205.61 | Panama | KX923820 | KX924254 | KX924044 | HG380462 |
|  | CBS 121662 | USA | KX923821 | KX924255 | KX924045 |  |
| ***Y. jiujiangensis*** | **JX11B02** | **GDMCC 3.1081** | **PP892051** | **PV131307** | **PV131306** | **PP930918** |
| *Y. penicillata* | CBS 130296 | China | JN831359 | KY659807 | KY659808 | KY659809 |
| *Y. smithii* | CBS 855.68 | Germany | KX923822 | KX924256 | KX924046 | KX924028 |
| *Microascus cirrosus* | CBS 462.97 | Netherlands | LN850782 | LN850879 | KX924147 | LN850831 |
